# *In Vitro* Mode of Action and Anti-thrombotic Activity of Boophilin, a Multifunctional Kunitz Protease Inhibitor from the Midgut of a Tick Vector of Babesiosis, *Rhipicephalus microplus*

**DOI:** 10.1371/journal.pntd.0004298

**Published:** 2016-01-08

**Authors:** Teresa C. Assumpção, Dongying Ma, Daniella M. Mizurini, R. Manjunatha Kini, José M. C. Ribeiro, Michail Kotsyfakis, Robson Q. Monteiro, Ivo M. B. Francischetti

**Affiliations:** 1 Vector Biology Section, Laboratory of Malaria and Vector Research (LMVR), National Institute of Allergy and Infectious Diseases (NIAID), National Institutes of Health (NIH), Bethesda, Maryland, United States of America; 2 Instituto de Bioquímica Médica Leopoldo de Meis, Universidade Federal do Rio de Janeiro, Rio de Janeiro, Brazil; 3 Protein Science Laboratory, Department of Biological Sciences, National University of Singapore, Singapore, Singapore; 4 Biology Centre of the Czech Academy of Sciences, Institute of Parasitology, České Budějovice, Czech Republic; University of California San Diego School of Medicine, UNITED STATES

## Abstract

**Background:**

Hematophagous mosquitos and ticks avoid host hemostatic system through expression of enzyme inhibitors targeting proteolytic reactions of the coagulation and complement cascades. While most inhibitors characterized to date were found in the salivary glands, relatively few others have been identified in the midgut. Among those, Boophilin is a 2-Kunitz multifunctional inhibitor targeting thrombin, elastase, and kallikrein. However, the kinetics of Boophilin interaction with these enzymes, how it modulates platelet function, and whether it inhibits thrombosis *in vivo* have not been determined.

**Methodology/Principal Findings:**

Boophilin was expressed in HEK293 cells and purified to homogeneity. Using amidolytic assays and surface plasmon resonance experiments, we have demonstrated that Boophilin behaves as a classical, non-competitive inhibitor of thrombin with respect to small chromogenic substrates by a mechanism dependent on both exosite-1 and catalytic site. Inhibition is accompanied by blockade of platelet aggregation, fibrin formation, and clot-bound thrombin *in vitro*. Notably, we also identified Boophilin as a non-competitive inhibitor of FXIa, preventing FIX activation. In addition, Boophilin inhibits kallikrein activity and the reciprocal activation, indicating that it targets the contact pathway. Furthermore, Boophilin abrogates cathepsin G- and plasmin-induced platelet aggregation and partially affects elastase-mediated cleavage of Tissue Factor Pathway Inhibitor (TFPI). Finally, Boophilin inhibits carotid artery occlusion *in vivo* triggered by FeCl_3_, and promotes bleeding according to the mice tail transection method.

**Conclusion/Significance:**

Through inhibition of several enzymes involved in proteolytic cascades and cell activation, Boophilin plays a major role in keeping the midgut microenvironment at low hemostatic and inflammatory tonus. This response allows ticks to successfully digest a blood meal which is critical for metabolism and egg development. Boophilin is the first tick midgut FXIa anticoagulant also found to inhibit thrombosis.

## Author Summary

Hematophagous animals express a repertoire of anti-hemostatics which target enzymes involved in proteolytic reactions. These molecules are present in the salivary glands or midguts and target components of both coagulation and complement cascades, in addition to cells involved in hemostasis and immune system. These inhibitors are critical for development and survival of mosquitoes and ticks, and might also contribute to parasite transmission and completion of their life cycle. While much is known regarding sialomics and functional genomics of the salivary glands components, comparatively less information has been gained over the years with respect to midgut anti-hemostatics and their mechanisms of action. The vector of Babesiosis and Q fever, *Rhipicephalus microplus*, expresses Boophilin, a midgut thrombin inhibitor with low specificity, which contributes to tick development. Notably, we reported that Boophilin targets FXIa, kallikrein, and neutrophil enzymes elastase and cathepsin G, which play a direct or indirect role in the contact pathway of the coagulation cascade. Boophilin also abrogates platelet aggregation by cathepsin G and plasmin, and attenuates Tissue Factor Pathway Inhibitor cleavage by elastase. *In vivo*, Boophilin inhibits thrombosis and promotes bleeding in mice. It is concluded that Boophilin redundantly down-modulates host biochemical reactions involved in mounting and sustaining pro-inflammatory events which are detrimental to tick development.

## Introduction

Hematophagous insects and ticks are strict blood feeders, which express protease inhibitors in different tissues and organs. These molecules belong to the Kunitz, Kazal, Serpin, Cathepsin, and Trypsin Inhibitor-Like (TIL)-domains family of enzyme inhibitors, among others, and prevent hemostasis, inflammation and immune responses. Most of these inhibitors have been identified in the salivary glands of hematophagous animals. They display unique specificity and high-affinity binding for coagulation factors, or components of the complement cascade. Protease inhibitors also affect immune cells, endothelial cells and platelet functions [1–10].

Other reports have provided evidence for expression of protease inhibitors in the midgut of blood-sucking animals. Accordingly, midgut thrombin inhibitors have been identified in *Ornithodorus moubatta* (ornithodorin) [11], *Haemaphysalis longicornis* (hemalin), *Triatoma brasiliensis* (brasiliensin) [12], *Dipetalogaster maxima* (dipetalogastin) [13] and *Rhodnius prolixus* (rhodniin) [14]. In addition, infestins family members from *Triatoma infestans* target thrombin, FXIIa or elastase and display antithrombotic activity *in vivo* [15–18] while rhiphilin-2 from *Rhipicephalus hemaphysaloides* inhibits elastase [19]. More recently, the two Kunitz-containing Boophilin from the tick *Rhipicephalus microplus* was found to block thrombin, but also interact with plasmin, elastase, kallikrein and Factor (F)VIIa [20,21]. The structure of Boophilin revealed that it inhibits thrombin in a non-canonical manner, despite possessing a canonical reactive site loop. Accordingly, residues in the N-terminal interacts with the catalytic site while the C-terminal Kunitz domain binds to the anion binding exosite-1 [20]. Functionally, RNAi silencing of Boophilin gene resulted in 20% less egg weight increase [21]. These results emphasize the importance of Boophilin in several aspects associated with tick feeding and metabolism. However, the kinetics of Boophilin interaction with distinct enzymes, how it modulates platelet function, and whether it inhibits thrombosis *in vivo* have not been determined.

## Materials and Methods

### Reagents

α-thrombin, γ-thrombin, and PPACK (Phe-Pro-Arg-chloromethylketone)-thrombin were from Hematologic Technologies (Essex Junction, VT). FXIa, FXIIa, and pre-kallikrein were from Enzyme Research Laboratories (South Bend, IN). FIX (Benefix, recombinant FIX, protein-free) was from Wyeth-Pfizer (New York, NY). APTT (STA-PTT Automate) and PT (Neoplastine CI Plus) reagents were from Diagnostica Stago (Asnieres, France). S2238 (H-D-phenylalanyl-L-pipecolyl-L-arginine-*p*-nitroanilinedihydrochloride), S2366 (L-pyroglutamyl-L-propyl-L-arginine*-p*-nitroanilide), and S2302 (H-D-L-prolyl-L-phenylanyl-L-arginine*-p*-nitroanilide) were obtained from Chromogenix-Diapharma (West Chester, OH). CM5 sensor chips and all reagents for surface plasmon resonance (BIAcore) experiments were from GE-Healthcare-Pharmacia (Piscataway, NJ). TFPI (human recombinant Tissue Factor Pathway Inhibitor) was from R&D Systems (Minneapolis, MN). Elastase (human neutrophil elastase) and human Cathepsin G were from Molecular Innovations. Dextran sulphate (DS) 500K was from Sigma Co. (Saint Louis, MO). Corn Trypsin Inhibitor (CTI) was from Kerafast Inc. (Boston, MA).

### Blood collection and ethics statement

Blood samples (human blood type O) were obtained from paid healthy volunteers who gave written informed consent to participate in an Institutional Review Board (IRB) approved protocol under the name “Collection and Distribution of Blood Components from Healthy Donors for In Vitro Research Use”. The protocol is designed to protect subjects from research risks as defined in 45CFR46 and to abide by all NIH guidelines for human subjects research (protocol number 99-CC-0168). Collection was performed at the NIH Department of Transfusion Medicine under the direction of Dr. Susan Leitman, as described. All animal care and experimental protocols were conducted following the guidelines of the institutional care and use committee (Committee for Evaluation of Animal Use for Research from the Federal University of Rio de Janeiro, CAUAP-UFRJ) and the NIH Guide for the Care and Use of Laboratory Animals (ISBN 0-309-05377-3). The protocols were approved by CAUAP-UFRJ under registry #IBQM/081-05/16. Technicians dedicated to the animal facility at the Institute of Medical Biochemistry (UFRJ) carried out all aspects related to mouse husbandry under strict guidelines to insure careful and consistent handling of the animals.

### Cloning and protein expression

Sequence for Boophilin reported in [21] and as TC4205 in [3] was synthesized by BioBasics (Ontario, Canada) and cloned in VR1020 vector, which contains the kanamycin-resistance gene, the cytomegalovirus promoter and the tissue plasminogen activator signal peptide. The signal peptidase cleavage site is preserved in this plasmid, and exhibits a *BamH*I restriction site in the 3’ end. Each synthetic sequence displays a *BamH*I restriction site (*Ggatcc*) at the 5’ end. A sequence coding the C-terminal of the protein, a stop codon, and a *BamH*I restriction site comprised the 3’ end. VR1020 plasmid was digested with the *BamH*I site followed by ligation. All plasmids were sequenced and found to be in frame with the insert and used for transformation of TOP10 cells as described. Mature proteins expressed with VR1020 vector reportedly contain additional residues in the N-terminus introduced by incorporation of the *BamH*I site [22]. Boophilin theoretical mass is 13992.35 *da* with a p*I* of 4.41. Plasmids were generated and used for transfection of human embryonic kidney 293-F cells at the Protein Expression Laboratory at NCI-Frederick (Frederick, MD). The supernatant was collected after 72 hours, centrifuged at 2000 rpm, and frozen.

### Protein purification

The supernatant containing Boophilin was concentrated from 500 to 30 mL using an ultrafiltration cell unit (Millipore, Billerica, MA) under continuous stirring and 40 mPa pressure with 10-kDa ultrafiltration membranes (Millipore). The concentrate was centrifuged to remove particles and dialyzed against 20 mM Tris-HCl, 0.15 M NaCl, pH 8.0 buffer (TBS). The sample was loaded in a Superdex G75 column equilibrated with the same TBS buffer. Elution was carried out at 1mL/min and active fractions for inhibition of Kallikrein assay (see below) were pooled. Then, 5% acetonitrile (ACN) was added to the pooled sample, which was acidified with trifluoracetic acid (TFA) 0.1%. Sample was loaded into a reverse-phase HPLC (Vydac, Carpenteria, CA) previously equilibrated in ACN 5%/TFA 0.1%. Elution was carried out at 1 mL/min using a 0–100% ACN, TFA 0.1% in 1 hour. Samples were dialyzed extensively against PBS, and frozen.

### SDS-PAGE

Samples were treated with 4× NuPAGE lithium dodecyl sulfate sample buffer and 10× sample reducing reagent, then loaded into NuPAGE-Bis-Tris 4% to 12% gels with 2-(N-morpholino)ethanesulfonic acid (MES) running buffer (Invitrogen). Gels were stained with Coomassie blue R-250.

### Tryptic digestion and mass spectrometry

The tryptic peptides spots were loaded on a Waters Nano acquity system (Waters, Milford, MA). The peptides were desalted on-line using a Waters Symmetry C18 180 μm X 20 mm, 5 μm trap column. The sample injection volume was typically 7.5 μl, and the LC was performed by using BEH 130 C18 100 μm X 100 mm, 1.7 μm column (Waters, Milford, MA) and eluting (0.5 μl/min) with a linear gradient (10–40%) of acetonitrile containing 0.1% formic acid. Electrospray tandem mass spectra were recorded using a Q-Tof quadrupole/orthogonal acceleration time-of-flight spectrometer (Waters, Milford, MA) interfaced to the Nano acquity system capillary chromatograph. The ESI voltage was set at 3500 V, the source temperature was 80°C and the cone voltage was 30 V. The instrument control and data acquisition were conducted by a MassLynx data system (Version 4.1, Waters), and experiments were performed by scanning from a mass-to-charge ratio (*m*/*z*) of 400–2000 using a scan time of 1 s, applied during the whole chromatographic process. The mass spectra corresponding to each signal from the total ion current (TIC) chromatogram were averaged, allowing for an accurate molecular mass measurement. The exact mass was determined automatically using the Q-Tof's LockSpray™ (Waters, Milford, MA). Data-dependent MS/MS acquisitions were performed on precursors with charge states of 2, 3 or 4 over a range of 50–2000 *m*/*z* and under a 2 *m*/*z* window. A maximum of three ions were selected for MS/MS from a single MS survey. Collision-induced dissociation (CID) MS/MS spectra were obtained using argon as the collision gas at a pressure of 40 psi, and the collision voltage was varied between 18 and 90 V depending on the mass and charge of the precursor. All data were processed using the ProteinLynx Global server (version 2.5, Waters). The processing automatically lock mass corrected the *m*/*z* scale of both the MS and MS/MS data utilizing the lock spray reference ion.

### CLUSTAL alignment and phylogenetic tree

A sequence similarity search to Boophilin from *Rhipicephalus microplus* was performed using protein BLAST. Some protein sequences were selected and aligned by the ClustalX software program: *R*. *microplus* (gi 74935652, Boophilin-H2; gi 74844209, Boophilin-G2), *D*. *variabilis* (gi 194246037), *H*. *longicornis* (gi 217034827) and *I*. *scapularis* (gi 240990666). The six letters derive from the first three letters of the genus and the first three letters from the species name. The phylogenetic tree was performed with the Mega package (Kumar et al. 2004) after 10,000 bootstraps with the neighbor joining (NJ) algorithm, using poisson model and pairwise deletion. The sequences from the nonreduntant (NR) protein database of the National Center for Biotechnology Information (NCBI) are represented by six letters followed by the NCBI gi| accession number.

### aPTT and PT assays

aPTT and PT were evaluated on a STart 4 stagocoagulometer (DiagnosticaStago, Parsippany, NJ). Freeze-dried, citrated, normal human plasma was suspended in ultra-pure water. For the aPTT, plasma (50 μL) was incubated with 1 μM Boophilin or PBS (control) in appropriate cuvettes and placed in the coagulometer for 5 minutes at 37°C. Then, 50 μL of pre-warmed aPTT reagent (STA PTT; DiagnosticaStago) was added and incubated for 3 more minutes. CaCl_2_ (50 μL at 25 mM) was added to start reactions. For the PT, plasma (50 μL) was incubated with 1 μM Boophilin or PBS (control) and placed in the coagulometer for 5 minutes at 37°C. Then, 100 μL of the PT reagent (NEOplastine CI plus; DiagnosticaStago) was added. Time for clot formation was recorded in duplicate.

### Kinetic studies

These were performed using chromogenic substrate hydrolysis (S2238 for thrombin; S2366 for FXIa; S2302 for Kallikrein) using a Thermomax ELISA microplate reader (Molecular Devices, Menlo Park, CA) as described [23–26]. All reagents were diluted in the reaction buffer, TBS-BSA (10 mM Tris, 0.15 M NaCl, 0.3% BSA, pH 7.4). Reactions were performed in 96-well flat-bottom plates (Corning 3596; Corning, NY) in a total volume of 200 μL. To characterize the interaction of Boophilin and thrombin or Boophilin and FXIa, reactions were started by addition of S2238 or S2366 (250 μM) to a mixture containing the enzyme (thrombin or FXIa) and inhibitor (0–640 nM) pre-incubated for 60 minutes at room temperature. Reactions were followed for 1 hour. For the assay designed to determine the type of inhibition (competitive or noncompetitive), the inhibitor (0–1280 nM) was incubated with S2238 or S2366 (125, 250, 500, and 750 μM) at room temperature for 5 minutes followed by addition of thrombin (0.2 nM) or FXIa (0.5 nM). Reactions were followed for 2 hours. In all kinetic measurements, care was taken to ensure that substrate was less than 20% hydrolyzed. The linear part of the progress curves between 30–60 minutes was chosen to determine the steady-state kinetics (*Vmax* mode) of Boophilin–thrombin or Boophilin–FXIa complex formation. The values for 1/V versus 1/S2238 or 1/S2366 were used to plot a double reciprocal (Lineweaver-Burk plot) for each Boophilin concentration, at a constant thrombin or FXIa concentration. In some experiments, Ki determination for tight inhibitor was used as described [26,27]. Data points were fitted for linear regression using GraphPad Prism software (GraphPad Software, Inc., San Diego, CA). Data points are the mean of 3 determinations, each performed in triplicate. To characterize the interaction of Boophilin and kallikrein, reactions were started by addition of S2302 (250 μM) to a mixture containing the kallilkrein and Boophilin (0–3200 nM) pre-incubated for 60 minutes at room temperature. Reactions were followed for 1 hour.

### Other chromogenic substrate hydrolysis

α-thrombin or γ-thrombin mediated hydrolysis of chromogenic substrate S2238 was evaluated in the presence of Boophilin at different concentrations (0 μM, 0.1 μM, 1 μM, 3 μM). Reactions started by addition of S2238 (final concentration: 250 μM) to a mixture containing Boophilin incubated with α-thrombin (0.2 nM) or γ-thrombin (0.2 nM) for 30 minutes. Substrate hydrolysis was followed at 37°C and 405 nm for 45 minutes. Kallikrein mediated hydrolysis of chromogenic substrate S-3202 was evaluated in the presence of the fractions obtained by purification in Superdex G75. Reactions started by addition of S3202 (250 μM) to mixture containing aliquots of each fraction incubated with Kallikrein. Substrate hydrolysis was followed at 37°C and 405 nm for 30 minutes.

### Protease inhibition assays

One hundred nM of Boophilin was pre-incubated with each enzyme for 20 minutes before addition of the corresponding substrate, as described [23,24,26,28]. Hydrolysis rate of the fluorescent substrate was estimated from the slope that results from the linear fit (arbitrary fluorescence units per sec, χ2 > 0.95) of the data (each experiment was performed in triplicate, at 30°C, and the mean of the three experiments and the standard error of the mean were calculated). The linear fit of the fluorescence increase as a function of time was verified with the Magellan™ –Data Analysis Software (Tecan group Ltd). The observed substrate hydrolysis rate in the absence of protein was considered as 100% and compared with the remaining enzymatic activity in the presence of the protein. All enzymes used were of human origin, purified or recombinant. The source and assay concentration of the different enzymes follow: thrombin (0.01 nM), α-chymotrypsin (0.025 nM), plasmin (1.6 nM), and chymase (0.9 nM), were purchased from Sigma; tryptase (0.009 nM) was purchased from Promega; FXa (0.33 nM) was purchased from EMD Biosciences (Madison, WI); FXIIa (0.1 nM) was purchased from Haematologic Technologies Inc.; kallikrein (0.04 nM) was purchased from Fitzgerald Industries International; elastase (0.036 nM) was purchased from Elastin Products; cathepsin G (6.6 nM), FXIa (0.06 nM), uPA (0.25 nM), and tPA (0.018 nM) were from Molecular Innovations; matriptase (0.3 nM) was from R&D Systems; proteinase 3 (9 nM) was from Merck; and sequencing-grade trypsin (0.1 nM) was purchased from Roche. Assay buffers were: for elastase, proteinase 3 and chymase, 50 mM Hepes buffer, pH 7.4, 100 mM NaCl, 0.01% Triton X-100; for trypsin, chymotrypsin, FXI and FXIIa, 50 mM Tris-HCl, pH 8.0, 150 mM NaCl, 20 mM CaCl_2_, 0.01% Triton X-100; for thrombin, 50 mM Tris-HCl, pH 8.0, 150 mM NaCl, 0.01% Triton X-100; for tryptase, 50 mM Tris-HCl, pH 8.0, 50 mM NaCl, 0.05% Triton X-100; for kallikrein, matriptase and plasmin, 20 mM Tris-HCl, pH 8.5, 150 mM NaCl, 0.02% Triton X-100; for FXa, 20 mM Tris-HCl, pH 8.0, 200 mM NaCl, 5 mM CaCl_2_, 0.1% BSA; for uPA and tPA, 20mM Tris-HCl, pH 8.5, 0.05% Triton X-100; and for cathepsin G, 50 mM Tris-HCl, pH 7.4, 150 mM NaCl, 0.01 Triton X-100. The substrates were Suc-Ala-Ala-Pro-Val-AMC for elastase and proteinase 3, Boc-Asp-Pro-Arg-AMC for thrombin and plasmin, Boc-Gln-Ala-Arg-AMC for trypsin, FXIa and uPA (Sigma), Boc-Phe-Ser-Arg-AMC for tryptase, Suc-Leu-Leu-Val-Tyr-AMC for chymase (Bachem Bioscience, Inc.), Suc-Ala-Ala-Pro-Val-AMC for chymotrypsin (EMD Biosciences) and methylsulfonyl-D-cyclohexylalanyl-Gly-Arg-AMC acetate for FXa, FXIIa, tPA, matriptase and kallikrein (American Diagnostica Inc.). All substrates were used in 250 μM final concentration in all assays. Substrate hydrolysis rate was followed in a Tecan Infinite M200 96-well plate fluorescence reader (Tecan group Ltd, Switzerland) using 365 nm excitation and 450 nm emission wavelengths with a cut-off at 435 nm. *t*-test was used for statistical analysis and a *p* value of 0.05 or less was considered statistically significant.

### Surface plasmon resonance

All surface plasmon resonance (SPR) experiments were carried out in a T100 instrument (Biacore Inc, Uppsala, Sweden) following the manufacturer’s instructions as reported [23,24,26]. For immobilization using an amine-coupling kit (Biacore), carboxymethylated dextran (CM5) chips were activated with 1-ethyl-3-(dimethylaminopropyl) carbodiimide and N-hydroxysuccinimide before injection of Boophilin (40 μg/mL) in acetate buffer, pH 4.0, or FXIa (10 μg/mL) in acetate buffer pH 5.0. Remaining activated groups were blocked with 1 M ethanolamine, pH 8.5, resulting in a final immobilization of 301.4 RU for Boophilin and 1280.4 RU for FXIa. Kinetic experiments were carried out by injecting thrombin over immobilized Boophilin for a contact time of 90 seconds, or by injecting Boophilin over immobilized FXIa for a contact time of 120 seconds, at a flow rate 30 μL/minute at 25°C. For all runs, HBS-P buffer (10 mM HEPES, 150 mM NaCl, 0.05% surfactant P20, pH 7.4) was used. Thrombin-Boophilin and FXIa-Boophilin complexes dissociation were monitored for 180 and 600 seconds, respectively. The sensor surface was regenerated by a pulse of 80 seconds of 1 mM HCl, 2 M NaCl at 30 μL/minute. In some experiments, α-thrombin, γ-thrombin, and PPACK-thrombin were tested (1 μM) as analytes. Blank flow cells were used to subtract the buffer effect on sensorgrams. After subtraction of the contribution of bulk refractive index and nonspecific interactions with the CM5 chip surface, the individual association (*ka or kon*) and dissociation (*kd or koff*) rate constants were obtained by global fitting of data using the 1:1 model (Langmuir) interaction model using BIAevaluation™ (Biacore, Inc.). Values were then used to calculate the equilibrium constant (*KD*). The values of average-squared residual obtained were not significantly improved by fitting data to models that assumed other interactions. Conditions were chosen so that the contribution of mass transport to the observed values of *KD* was negligible. Also, models in the T100 evaluation software fit for mass transfer coefficient to mathematically extrapolate the true *ka* and *kd*.

### Platelet aggregation

Plasma rich-plasma (PRP) was obtained by plateletpheresis from medication-free platelet donors at the DTM/NIH blood bank as described [29]. PRP was centrifuged at 1,100 x g for 15 minutes and washed once in Tyrode Buffer (5 mM HEPES, 137 mM NaCl, 2.7 mM KCl, 12 mM NaHCO_3_, 0.42 mM Na_2_HPO_4_, 1 mM MgCl_2_, 0.25% BSA, 5.55 mM glucose, pH 7.4), supplemented with apyrase (final concentration: 0.4 U/mL) and EDTA (final concentration: 5 mM). Platelets were centrifuged again and suspended in Tyrode buffer only. Aggregation was performed as described in [29]. Tyrode buffer (270 μL) was added to 30 μL of platelets to give a final concentration of 200,000 to 400,000 platelets/μL. Platelets were placed in a cuvette and stirred at 1,200 rpm at 37°C for 1 minute, followed by addition of reagents, indicated by the figure legends. Aggregation response was monitored by turbidimetry using a Lumi-Aggregometer (Chrono-Log Corp).

### Clot-bound α-thrombin

Fibrin clots were prepared by incubation of 300 μL of fibrinogen (2 mg/mL in 50 mM HEPES, pH 7.5, 150 mM NaCl, 10 mM CaCl_2_, 1 mg/mL PEG) with 30 nM α-thrombin for 2 hours at 37°C as described [25]. Clots were washed 8 times in the same buffer and transferred to another tube. Clots were incubated with 200 μL of solution containing different concentrations of Boophilin (diluted in 50 mM HEPES, pH 7.5, 150 mM NaCl, 10 mM CaCl_2_, 1 mg/mL PEG) for 30 minutes at 37°C. The chromogenic substrate S-2238 was added (final concentration: 200 μM) and incubated for 90 minutes at 37°C. Aliquots were taken and substrate hydrolysis was estimated by end point reading at 405nm.

### Fibrinogen clotting assay

Thrombin (200 nM) was incubated with different concentrations of Boophilin (0 μM, 0.25 μM, 0.5 μM, 1 μM, and 3 μM) in 20 mM Tris-HCl, pH 7.5 for 10 minutes at 37°C [25]. Fibrinogen (final concentration: 2 mg/mL) was added, to a final volume of 100 μL. Absorbance at 600 nm was recorded for 30 minutes, at 30 seconds intervals.

### FIX activation by FXIa

In all experiments, plasma- and albumin-free recombinant FIX was used (BeneFIX, 250 U diluted at 1 U/μL in distilled water) [23]. In a final volume of 25 μL, FXIa (3 nM) was incubated for 20 minutes with Boophilin (0.3 μM, 1.0 μM or 3.0 μM) followed by addition of recombinant FIX (1 μM). After 60 minutes at 37°C, reactions were stopped by addition of Laemmli buffer and boiling with dithiothreitol for 5 minutes. Proteins were separated by 4% to 12% NuPAGE (MES buffer). Gel was stained with Coomassie Blue R-250.

### Reciprocal activation

Factor XII (0.2 nM final concentration) was preincubated with Boophilin (0 nM, 125 nM, 250 nM, 500 nM, 1000 nM, and 2000 nM) in 20 mM Tris, 0.15 M NaCl, 0.3% BSA, pH 7.4, for 20 minutes at room temperature [23,24]. Reciprocal activation was started by addition of pre-kallikrein (10 nM) and DS 500 (0.2 μg/mL). After 10 minutes incubation, substrate S-2302 (250 μM) was added, and the increase in absorbance at 405 nm was recorded at time intervals of 1 minute. Background hydrolysis of S-2302 by FXIIa was minimal and precluded the use of CTI to inhibit FXIIa.

### TFPI cleavage

Boophilin at different concentrations (0 μM, 1 μM, and 3 μM) was incubated with 1 μg of TFPI, in a final volume of 20 μL, in the presence of elastase (final concentration: 0.8 μg/mL), for 2 hours at room temperature [30]. Reactions were stopped by addition of Laemmli buffer and boiling with dithiothreitol for 5 minutes. Proteins were separated by 4% to 12% NuPAGE (MES buffer) and gel was stained with Coomassie Blue R-250.

### FeCl_3_-induced artery thrombosis

BALB/c mice were anesthetized with intramuscular xylazine (16 mg/kg) followed by ketamine (100 mg/kg) [23,26,30,31]. The right common carotid artery was isolated through a midline cervical incision, and blood flow was continuously monitored using a 0.5-VB Doppler flow probe coupled to a TS420 flow meter (Transonic Systems, Ithaca, NY). Fifteen minutes before induction of thrombosis, animals were injected in the tail vein with 50 μL Boophilin (0.1 or 1 mg/kg) or vehicle (PBS). Thrombus formation was induced by applying a piece of filter paper (1×2 mm) saturated with 5% FeCl_3_ solution on the adventitial surface of the artery for 3 minutes. After exposure, the filter paper was removed, and the vessel was washed with sterile normal saline. Carotid blood flow was continuously monitored for 60 minutes or until complete occlusion (0 flow for at least 10 seconds) occurred. Statistical analysis of variance using Tukey as a multiple comparison post-test was used. *p* ≤ 0.05 was considered statistically significant.

### Tail bleeding assay

Mice were anesthetized with intramuscular xylazin (16 mg/kg) followed by ketamine (100 mg/kg) and injected intravenously with phosphate-buffered saline (PBS) or Boophilin (1.0 mg/kg) in a 100 μL volume [23,26,30,31]. After 15 minutes, the distal 2 mm segment of the tail was removed and immediately immersed in 40 mL distilled water warmed to 37°C. The samples were properly homogenized and the absorbance determined at 540 nm to estimate hemoglobin content. No animal was allowed to bleed for more than 30 min.

## Results

**Fig 1:**
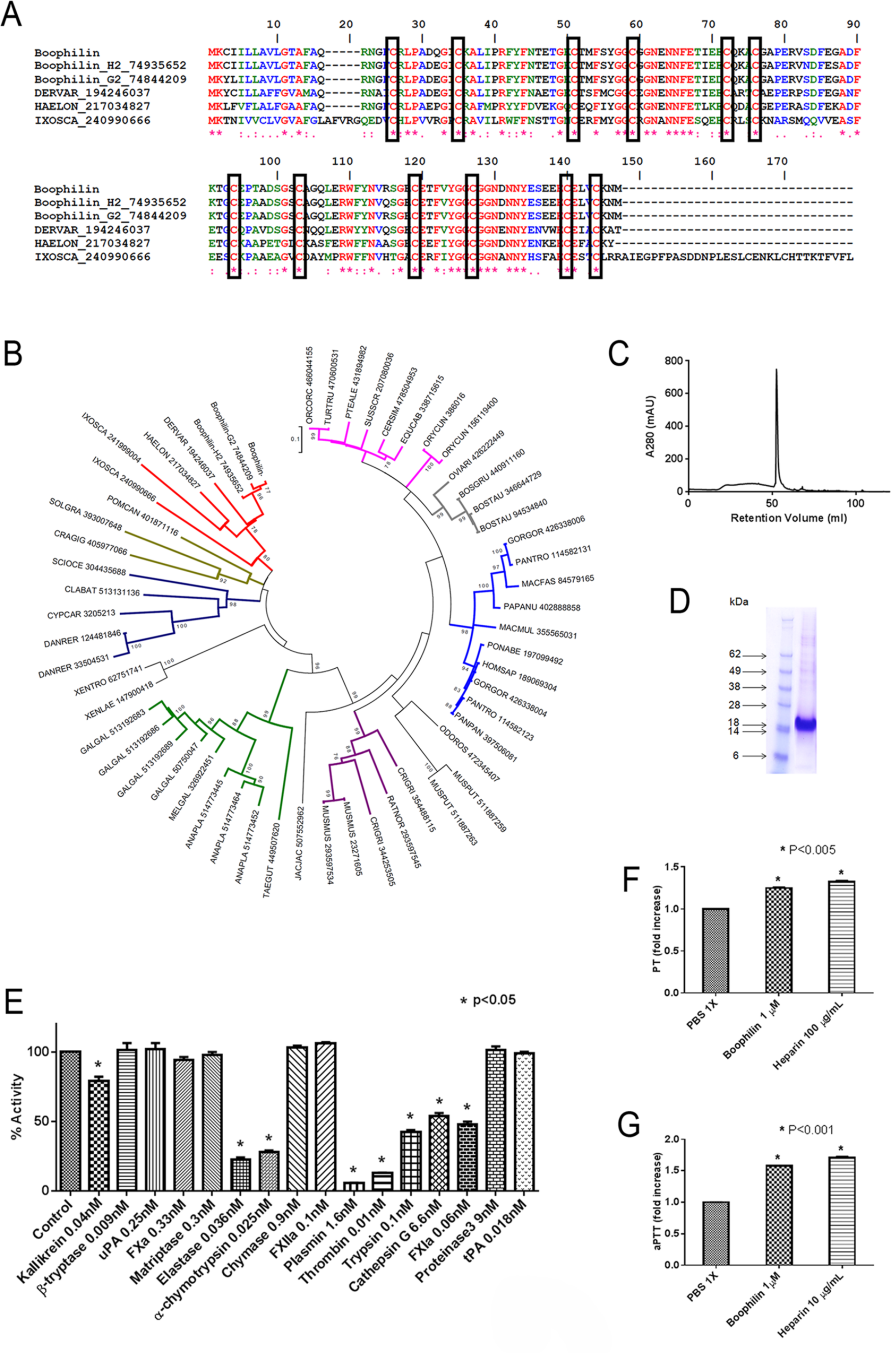
Characterization of Boophilin. (A) Clustal alignment of Boophilin [3,21] with other proteins of the Kunitz family. The boxes indicate the 12 conserved cysteines. The symbols below the alignment indicate: (*) identical sites; (:) conserved sites; (.) less conserved sites. (B) Phylogram of Boophilin and other Kunitz proteins, obtained by the Neighbor-joining algorithm, using pairwise deletion and poisson model. The sequences from the nonreduntant (NR) protein database of the National Center for Biotechnology Information (NCBI) are represented by the first 3 letters of their genus name, followed by the first three letters of the species name, followed by their gi| accession number. The numbers in the phylogram nodes indicate percent bootstrap support for the phylogeny after 10,000 iterations. The bar indicates 10% amino acid divergence in the sequences. (C) Reverse-phase chromatography of Boophilin expressed in HEK293 cells. (D) Boophilin was loaded in a NuPAGE gel under reducing conditions. Gel was stained with Coomassie Blue. On the left, molecular mass markers are indicated. (E) Protease screening. Screening for inhibition of several enzymes by Boophilin (100 nM): catalytic activity was estimated by fluorogenic substrate hydrolysis. (F) PT. Boophilin at 1 μM was added to the plasma, followed by addition of PT reagent as described in Methods. PT time, control human plasma: 17.5±0.25 seconds. (G) aPTT. Boophilin at 1 μM was added to the plasma, followed by addition of aPTT reagent as described in Methods. aPTT, control human plasma: 44.9±0.85 seconds. Clotting was estimated using a coagulometer and heparin was used as a control.

Fig 1A shows the alignment for Boophilin sequence employed for this study and elsewhere [3,21], and the 2 variants deposited in the databases: Boophilin-G2 (gi 74844209) and Boophilin-H2 (gi74935652). Fig 1B shows a phylogenetic tree constructed with the sequences of several Kunitz protease inhibitors from other species. Boophilin clades with orthologous from *Dermacentor* sp (gi 194246037), *Hyalomma* sp (gi 217034827), and *Ixodes* sp (gi 241999004) indicating that it is belongs to an expanded family of proteins.

Boophilin was expressed in HEK293 cells, and purified through gel-filtration and reverse phase chromatography in a C18 column (Fig 1C). Recombinant Boophilin was detected in each fraction through inhibition of the amidolytic activity of kallikrein. SDS-PAGE of Boophilin shows that it migrates as a single band with *ml wt* slightly larger than calculated molecular mass of ~ 14 kDa (Fig 1D). This discrepancy is likely due to potential n’ glycosylation sites in Asn positions 18, 59, 108 and 127 where n' represents an Asn with a positive score, but not occurring within an Asn-Xaa-Ser/Thr sequon (NetNGlyc 1.0 Server). Edman degradation determined that the N-terminus of Boophilin is blocked (Signal P server). Confirmation of Boophilin identity was obtained by tryptic digestion and mass-spectrometry, with coverage of above 40%. The following peptides were identified: FYFNTETGK, ACGAPERVSDFEGADFK and SGECETFVYGGCGGNDNNYESEEECELVCK. The specificity of Boophilin was tested in a screening assay optimized to detect protease inhibitors, as described in Methods. Fig 1E shows that Boophilin blocks partially or entirely the amidolytic activity of FXIa, cathepsin G, trypsin, thrombin, plasmin, chymotrypsin, elastase, and kallikrein. In contrast, Boophilin does not affect enzymatic activity of FXIIa, β-tryptase, uPA, FXa, matryptase, chymase, and proteinase-3. Consistent with these findings, Boophilin (1 μM) prolongs PT ~ 1.2 (Fig 1F) and aPTT ~ 1.5 fold (Fig 1G). Inhibition was comparable to prolongation produced by heparin at 10 μg/ml (aPTT), or at 100 μg/ml (PT).

**Fig 2:**
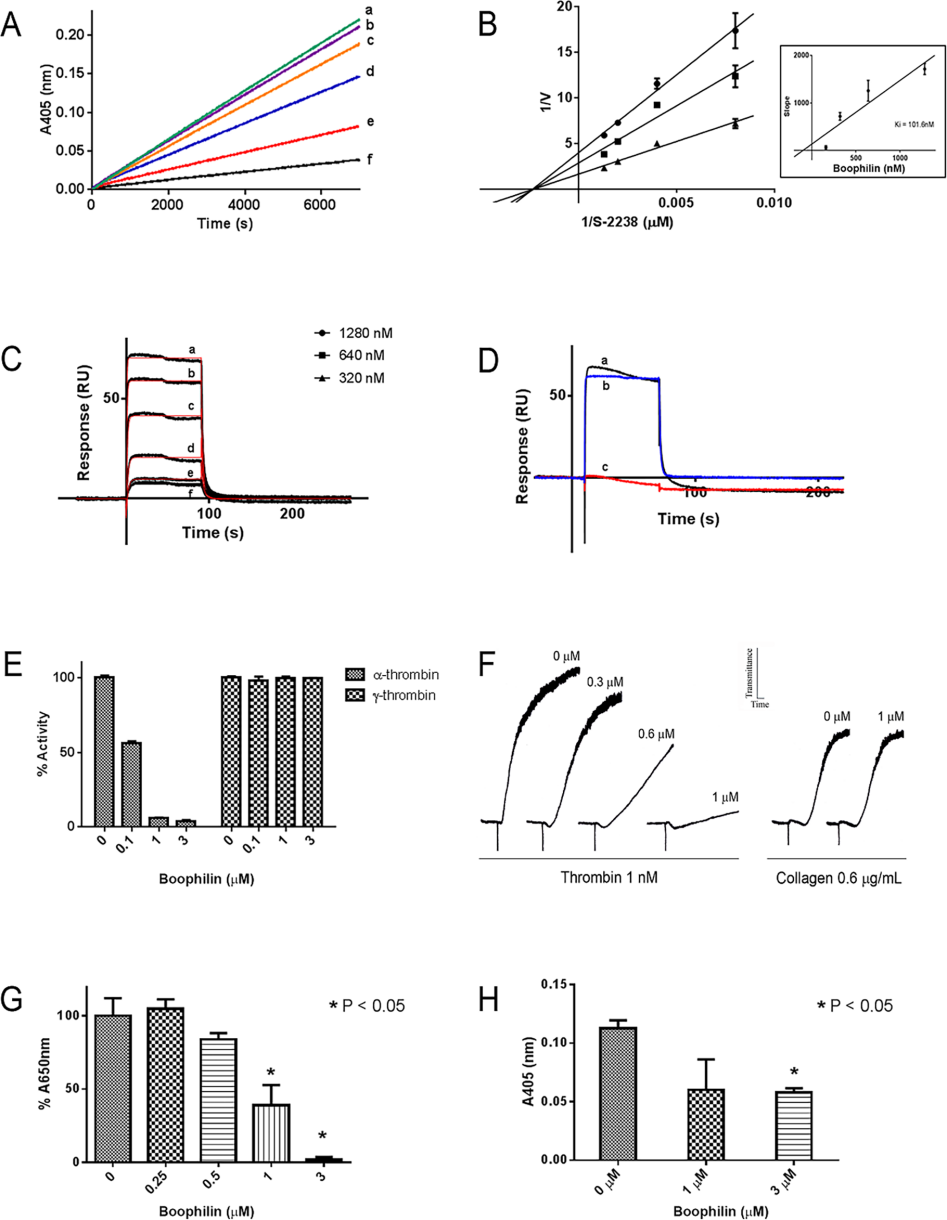
Boophilin inhibits thrombin. (A) Typical progress curves for thrombin-mediated hydrolysis of S2238 in the absence and presence of Boophilin at the indicated concentrations (a, 0 nM; b, 20 nM; c; 40 nM; d, 80 nM; e, 160 nM; f, 320 nM). Reactions started with addition of S2238 (250 μM) to a mixture containing Boophilin incubated with thrombin (0.2 nM) for 30 minutes. Substrate hydrolysis was followed for 2 hours at 37°C and 405 nm. (B) Lineweaver-Burk plot of the inhibition of thrombin by Boophilin (320 nM, 640 nM, and 1280 nM) at different concentrations of substrate S2238. Inset: Re-plot of the slope versus Boophilin concentration. (C) SPR experiments. Thrombin at the indicated concentrations (a, 500 nM; b, 250 nM; c; 125 nM; d, 62.5 nM; e, 31.25 nM; f, 15.625 nM) was injected over immobilized Boophilin for 90 seconds. Dissociation of the Boophilin-thrombin complex was monitored for 180 seconds, and a global 1:1 binding model was used to calculate kinetics parameters. Representative sensorgrams are shown in black lines and fitting of the data points using the Langmuir equation is depicted in red lines. (D) Boophilin binds to α-thrombin (a) and PPACK-thrombin (b) but does not interact with γ-thrombin (c). All the analytes were tested at 1 μM and injected in a sensor chip containing immobilized Boophilin. (E) α-thrombin or γ-thrombin mediated hydrolysis of S2238 in the presence of Boophilin at the indicated concentrations. Reactions started with addition of S2238 (250 μM) to a mixture containing Boophilin incubated with α-thrombin (0.2 nM) or γ-thrombin (0.2 nM) for 30 minutes. Substrate hydrolysis was followed at 405 nm. (F) Platelet aggregation. Washed platelets were stimulated by thrombin (1 nM) in the absence or presence of Boophilin (0.3 μM, 0.6 μM, and 1.0 μM). Control: collagen-induced platelet aggregation. (G) Fibrinogen clotting activity. Thrombin (200 nM) was incubated with different concentrations of Boophilin for 10 minutes at 37°C. Fibrinogen (final concentration: 2 mg/mL) was added and absorbance at 600nm was recorded for 30 minutes, at 30 seconds intervals. (H) Clot-bound α-thrombin. Fibrin clots were incubated with 1 μM and 3 μM of Boophilin or PBS 1X. Chromogenic substrate (S2238) hydrolysis was estimated by end point reading at 405nm.

Fig 2A shows the progress curves for thrombin inhibition in the presence of increasing concentration of Boophilin. Double-reciprocal plots and transformation of the data determined that Boophilin behaves as a classical non-competitive inhibitor of thrombin (Fig 2B), with a calculated *Ki* 101 nM (Fig 2B, inset). Plasmon resonance experiments (SPR) were employed as a second method to study kinetics. Fig 2C depicts typical sensorgrams for Boophilin-thrombin interactions yielding a *kD* 116 nM (*kon* 3 x 10^6^ M^-1^.s^-1^; *koff* 0.3603 s^-1^). It has been reported that thrombin function is dependent on the catalytic site, and the exosite which mediate enzyme interaction with distinct physiological substrates and inhibitors [32]. Two variants of thrombin were used in this study, including catalytic site-blocked PPACK-thrombin, and exosite-1 deleted γ-thrombin. SPR experiments show that PPACK-thrombin reacts with immobilized Boophilin in a similar fashion as native thrombin (Fig 2D). On the other hand, γ-thrombin interaction is completely blunted by removal of exosite-1, suggesting an important role for this domain. Inhibition of thrombin variants by Boophilin were also tested functionally. Fig 2E shows that the amidolytic activity of α-thrombin was dose-dependently blocked by Boophilin; however, no inhibition was observed with γ-thrombin. Functionally, thrombin- but not collagen-induced platelet aggregation was abrogated by Boophilin, with maximum effect observed at 1 μM (Fig 2F). Boophilin also attenuated thrombin-mediated fibrin formation *in vitro*, with an *IC*_*50*_ ~ 1 μM (Fig 2G). Clot-bound thrombin is an important component of thrombus formation, leading to propagation of enzymatic reactions. It is also a potential limitation for some anticoagulants which do not inhibit fibrin-bound thrombin [32]. The effect of Boophilin in clot-bound thrombin was detected as residual amidolytic activity associated with fibrin, using S-2238. Fig 2H shows that Boophilin attenuates clot-bound thrombin with an *IC*_*50*_ ~ 1 μM.

**Fig 3:**
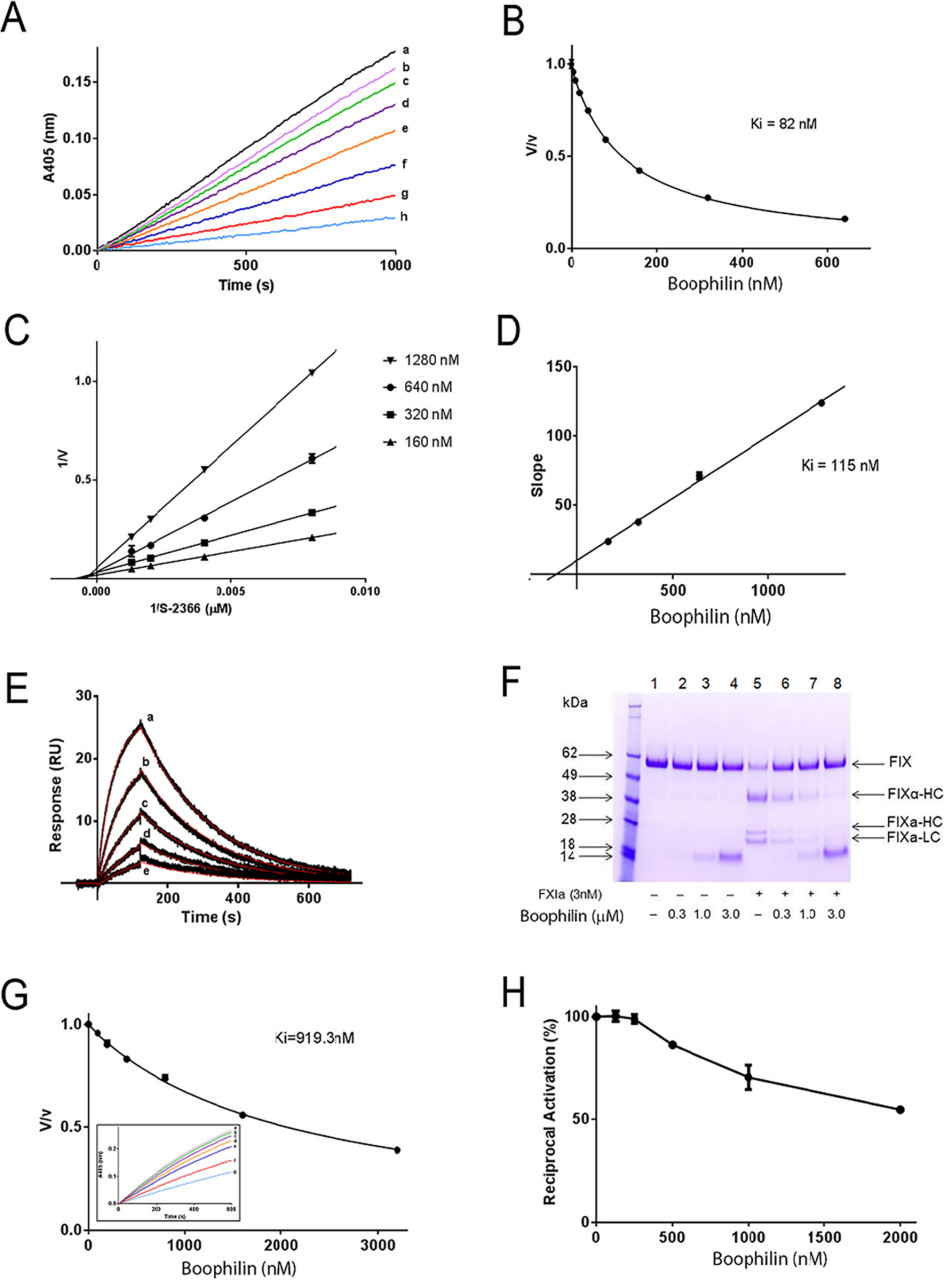
Boophilin inhibits FXIa and Kallikrein. (A) Typical progress curves for FXIa-mediated S2366 hydrolysis in the absence and presence of Boophilin at the indicated concentrations (a, 0 nM; b, 10 nM; c, 20 nM; d, 40 nM; e, 80 nM; f, 160 nM; g, 320 nM; h, 640 nM). Reactions started with addition of S2366 (250 μM) to a mixture containing Boophilin incubated for 1 hour with FXIa (10 nM). Substrate hydrolysis was followed for 2 hours at 37°C and 405 nm. (B) The ratio of *Vs*/*Vo in* (A) was plotted against Boophilin concentration to calculate the apparent *Ki*. (C) Lineweaver-Burk plot of the inhibition of FXIa activity by Boophilin (160 nM, 320 nM, 640 nM, and 1280 nM) at different concentrations of substrate S2366. (D) Re-plot of the slope from Lineweaver-Burk plot versus Boophilin concentrations. (E) SPR experiments. Boophilin at different concentrations (a, 500 nM; b, 250 nM; c; 125 nM; d, 62.5 nM; e, 31.25 nM) was injected over immobilized FXIa for 120 seconds. Dissociation of the Boophilin-FXIa complex was monitored for 600 seconds, and a global 1:1 binding model was used to calculate kinetics parameters. Representative sensorgrams are shown in black lines, and fitting of the data points using the Langmuir equation is depicted in red lines. (F) Inhibition of FIX activation. Control (lanes 1, 2, 3, 4): recombinant FIX (BeneFIX, 1.0 μM) in the presence of PBS (lane 1) or indicated concentrations of Boophilin (lanes 2, 3, 4). Lanes 5, 6, 7 and 8: FXIa (3 nM) was added to FIX in the presence of PBS (lane 5) or Boophilin (lanes 6, 7, 8), and mixture was incubated at 37°C for 60 min. Reactions were stopped with reducing Laemmli buffer, and proteins were separated by 4% to 12% NuPAGE. The bands correspond to (from the top): uncleaved FIX (FIX), the heavy chain FIXα (FIXα-HC), the heavy chain of FIXa (FIXa-HC), and the light chain of FIXa (FIXa-LC). (G) The ratio of kallikrein activity (*Vs*/*Vo)* was plotted against Boophilin concentration. *Inset*: typical progress curves for kallikrein-mediated hydrolysis of S2302 in the absence and presence of Boophilin at the indicated concentrations (a, 0 nM; b, 100 nM; c; 200 nM; d, 400 nM; e, 800 nM; f, 1600 nM; g, 3200 nM). Reactions started with addition of S2302 (250 μM) to a mixture containing Boophilin incubated with kallikrein (2 nM) for 1 hour. Substrate hydrolysis was followed for 2 hours at 37°C and 405 nm. (H) Reciprocal activation. Factor XII (0.2 nM) was preincubated with Boophilin (0 nM, 125 nM, 250 nM, 500 nM, 1000 nM and 2000 nM) in 20 mM Tris, 0.15 M NaCl, 0.3% BSA, pH 7.4, for 10 minutes at room temperature. Reactions were started by addition of pre-kallikrein (10 nM) and DS 500 (0.2 μg/mL, final concentrations). Data points are the mean+/-SD (n = 3).

FXIa is an important enzyme in the contact pathway, and plays a major role in thrombus formation *in vivo*. It is an attractive target since its inhibition is not associated with a bleeding phenotype [33]. Fig 3A shows progress curves of FXIa amidolytic activity inhibition by Boophilin. Transformation of the data as *Vs* (final velocity)/*Vo*(initial velocity) yields an apparent *Ki* ~ 82 nM (Fig 3B). Double-reciprocal plots determined that the inhibition is most compatible with a non-competitive type (Fig 3C) with a calculated *Ki* 115 nM (Fig 3D). Next, SPR experiments were performed. Fig 3E shows typical sensorgrams depicting the kinetics of FXIa/Boophilin interactions, with a calculated *kD* ~ 125 nM. Functionally, inhibition of FXIa by Boophilin was estimated using FXIa-dependent activation of FIX. Upon cleavage, FIX is converted to FIXα-HC, FIXa-HC, and FIXa-LC, with corresponding bands being detectable by SDS-PAGE [33]. Fig 3F shows that Boophilin blocks FIX activation by FXIa, suggesting formation of a bi-molecular inhibitory complex, which prevents enzyme interaction with a main physiological substrate. FXIa and kallikrein display high sequence homology, with the distinction that FXIa is a homodimer. Kallikrein also plays an important role in contact pathway activation, and inflammation [33]. Fig 3G and inset show that Boophilin partially inhibits the amidolytic activity of kallikrein, with an *IC*_*50*_ 919 μM. Since prekalikrein and FXII are reciprocally activated, the effect of Boophilin was tested in this reaction. Fig 3H depicts that Boophilin partially prevents reciprocal activation, as expected for a kallikrein inhibitor.

**Fig 4:**
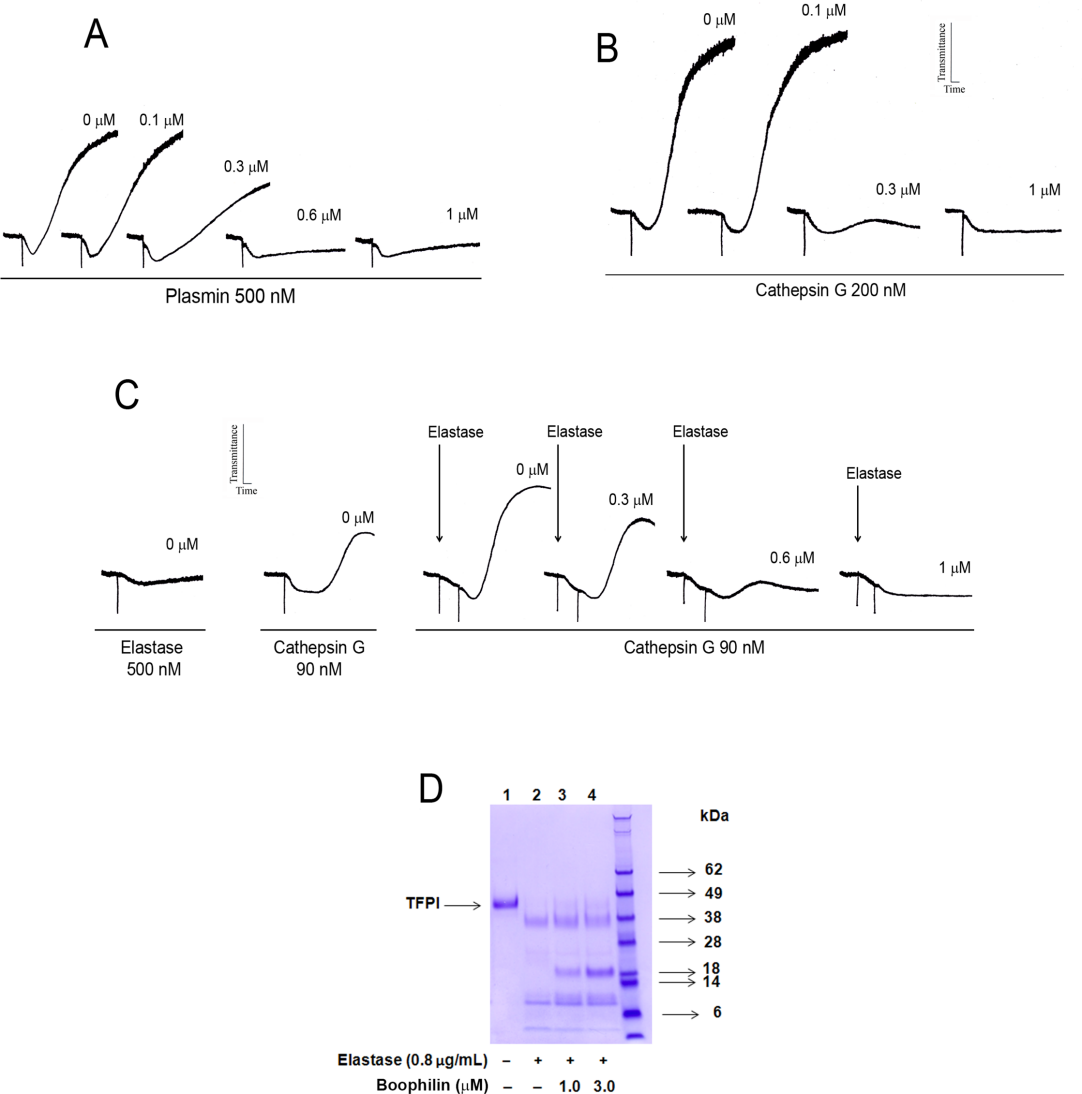
Boophilin inhibits plasmin, cathepsin G-induced platelet aggregation and elastase-mediated cleavage of TFPI. (A) Washed platelets were stimulated by plasmin (500 nM) in the absence or presence of Boophilin (0.1 μM, 0.3 μM, 0.6 μM, and 1.0 μM). (B) Washed platelets were stimulated by Cathepsin G (200 nM) in the absence or presence of Boophilin (0.1 μM, 0.3 μM, and 1.0 μM). (C) Washed platelets were induced to aggregate by Elastase (500 nM) and Cathepsin G (90 nM) with the indicated concentrations of Boophilin. (D) TFPI Cleavage. Boophilin (0 μM, 1 μM, and 3 μM) was incubated with TFPI (1 μg), in the presence of elastase (final concentration: 0.8 μg/mL), for 2 hours at room temperature. Reactions were stopped by addition of Laemmli buffer and boiling with dithiothreitol for 5 minutes. Proteins were separated by 4% to 12% NuPAGE (MES buffer) and gel was stained with Coomassie Blue R-250. Control: TFPI only.

Platelets play a major role in thrombus formation, and inflammatory reactions [34]. Several proteases besides thrombin positively-modulate platelet activation, including plasmin, cathepsin G, and elastase [35,36]. These enzymes have several targets in addition to platelets. For example, plasmin activates the complement cascade, and immune cells, besides modulation of fibrinolysis [37]. Neutrophil enzymes such as elastase and cathepsin G participate in thrombosis, through release of NETs, production of free radicals, and cleavage of TFPI [38–40]. Our results show that Boophilin is a plasmin inhibitor, according to inhibition of platelet aggregation (Fig 4A). Boophilin also dose-dependently inhibits platelet aggregation induced by high doses of cathepsin G (Fig 4B), or by lower doses of the enzyme in the presence of potentiating concentrations of elastase (Fig 4C). In addition, Boophilin partially (~20%) interferes with TFPI cleavage by elastase (Fig 4D).

**Fig 5:**
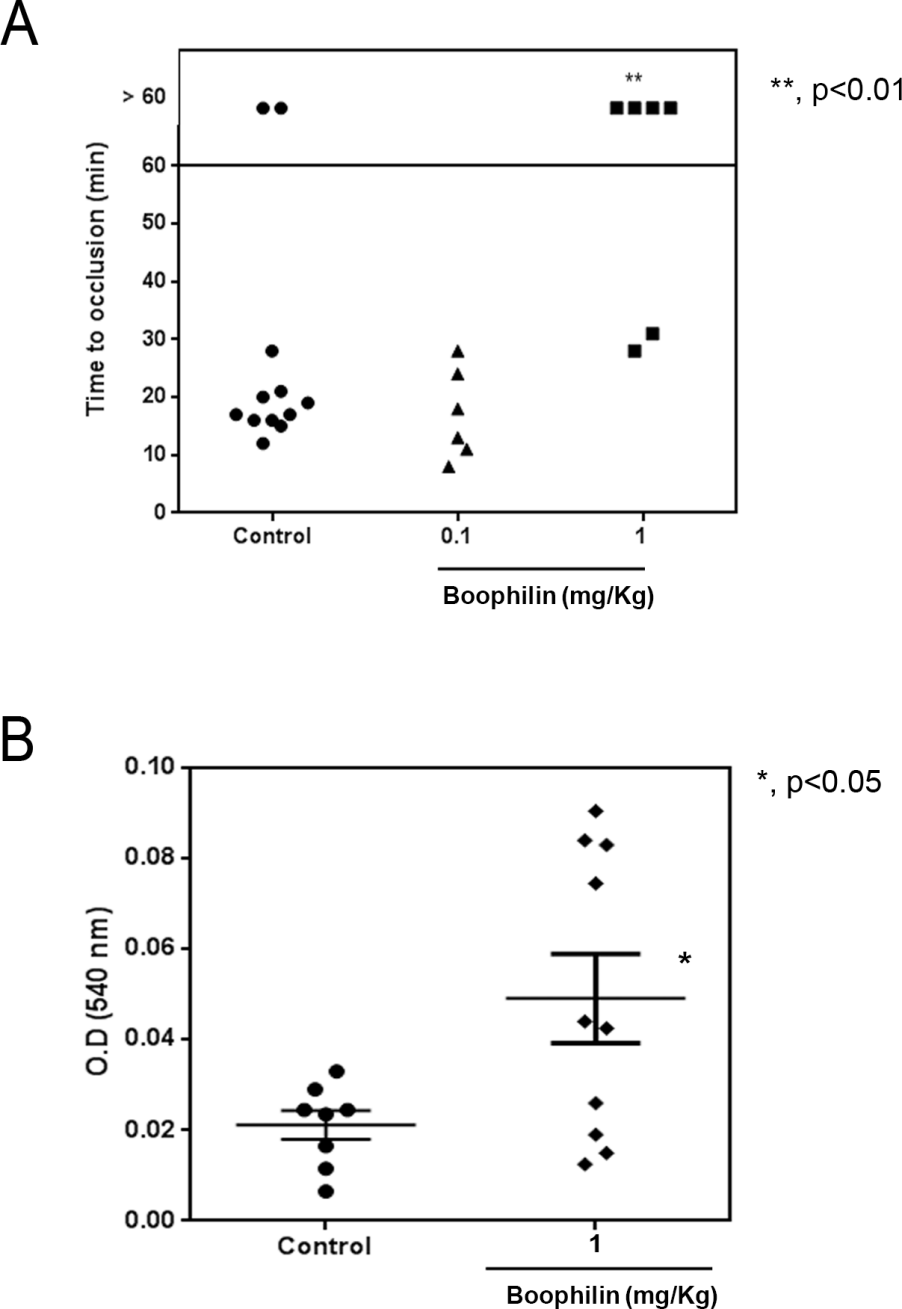
Boophilin inhibits thrombosis *in vivo*. (A) Thrombosis *in vivo*. A paper filter imbibed with 5% FeCl_3_ was applied to the carotid artery, and blood flow was monitored with a perivascular flow probe for 60 minutes or until stable occlusion took place. Fifteen minutes before injury, Boophilin was injected into the caudal vein of the mice. Each symbol represents one individual. **, *p* < 0.01. (B) Bleeding time. Bleeding was caused by a tail transection after *i*.*v*. injection of Boophilin at 1 mg/Kg. Absorbance at 540 nm was used to estimate blood loss. *, *p*<0.05 (ANOVA, with Dunnett post-test).

The effect of Boophilin *in vivo* was tested using the FeCl_3_-induced thrombus formation. This experimental model has been useful to determine the antithrombotic activity of several salivary anti-hemostatics [23,26,30,31,41]. Fig 5A shows that the occlusion time in control animals is ~ 18 min. Boophilin at 0.1 mg/Kg does not affect thrombus formation; however, at 1 mg/Kg Boophilin carotid occlusion did not take place for >60 min, in most animals. Consistent with anticoagulant activity, Boophilin at 1 mg/Kg promoted bleeding, according to the tail transection model (Fig 5B).

## Discussion

During feeding, blood remains in fluid phase in the mouthparts and digestive tracts of hematophagous arthropods. Protease inhibitors are critical in this step, since it interferes with several pro-hemostatic enzymes involved in clot formation and inflammation. Most inhibitors reported to date have been discovered in the salivary glands [1–10]. Usually, these molecules exhibit high-affinity binding and specificity towards components of the coagulation cascade, and complement system or interfere with different cell types involved in inflammatory and immune responses [4]. In contrast, fewer inhibitors have been comparatively found in the midgut of these animals, including hemalin from *Haemaphysalis longicornis* [12] and infestin family members from *Triatoma* infestans which target FXIIa, elastase and thrombin [15–17]. Thrombin inhibitors have also been identified in *Ornithodorus moubatta* (ornithodorin) [11], *T*. *brasiliensis* (brasiliensin) [12], *R*. *hemaphysaloides* (rhipilin-2)[19], *Dipetalogaster maxima* (dipetalogastin) [13] and *Rhodnius prolixus* (rhodniin) [14]. More recently, Boophilin from the midgut of *Riphicephalus microplus* was characterized as a multifunctional protease inhibitor affecting thrombin, plasmin and elastase, with residual activity towards kallikrein and FVIIa [4,21]. Boophilin inhibits thrombin in a non-canonical manner, despite possessing a canonical reactive site loop [20]. The importance of Boophilin has been demonstrated by silencing Boophilin gene, which resulted in 20% reduction in egg weight production [21]. This effect might be explained by inhibition of midgut proteases initiating and sustaining inflammatory reactions, which are presumably detrimental to tick and mosquito survival [42,43].

Several unknown aspects of Boophilin have been elucidated in this study, with respect to kinetics, effects on platelets, and *in vivo* anti-thrombotic activity. Boophilin expressed in HEK293 cells behaves as a classical, non-competitive inhibitor of thrombin with respect to small chromogenic substrate, with a *Ki* ~ 100 nM (amidolytic assays and SPR experiments). This value is higher than the previously calculated *Ki* 50 pM [21], and *Ki* 1800 pM [20]. The reasons for these incongruent values reported in our study and elsewhere [20,21] might be related to additional residues in the N-terminus introduced by the BamH1 required for cloning in the VR10/20 vector [22] and/or post-translation modifications such as glycosylation. Nevertheless, inhibition of thrombin function in all three studies, verified here and elsewhere [20] by prolongation of the PT or aPTT, or by inhibition of thrombin enzymatic activity using fluorogenic substrates [21], was achieved at relatively similar high concentrations (0.4–4.2 μM). In our study, we also demonstrated that Boophilin inhibits thrombin-induced platelet aggregation and fibrin formation, *in vitro*. These results are important since thrombin cleaves Protease-Activated Receptors (PARs) leading to cell activation and release of inflammatory cytokines [44]. We also found out that Boophilin does not inhibit the amidolytic activity of γ-thrombin; nor it interacts with γ-thrombin by SPR. On the other hand, PPACK-thrombin retains its ability to interact with the inhibitor. It is plausible that exosite-1 contributes extensively to enzyme-inhibitor complex formation, with participation of the catalytic site. These results are also supported by the crystal structure determination of Boophilin structure which revealed that residues in the N-terminal interacts with the catalytic site of thrombin while the C-terminal Kunitz domain binds to the anion binding exosite-1 [20].

Our results also indicate that Boophilin affects the contact pathway through different mechanisms: *i*) Boophilin is a classical and non-competitive inhibitor of FXIa with a Ki ~ 100 nM. Inhibition of FXIa was accompanied by blockade of FIX activation, providing direct evidence that Boophilin blocks the coagulation cascade by interfering with FIXa production. These results were also consistent with prolongation of the aPTT. The finding that Boophilin interferes with FXIa is relevant and novel, since this enzyme and others of the intrinsic pathway, (*e*.*g*. FIXa) are critical for contact activation, and thrombus formation *in vivo*, with minimal effect on hemostasis [31,33]. *ii*) Boophilin also partially prevents kallikrein amidolytic activity, and the reciprocal activation without affecting FXIIa. It is concluded that Boophilin interacts productively with kallikrein to prevent inflammatory reactions. *iii*) Platelets play a critical role in hemostasis, and several proteases besides thrombin are known to modulate platelet function and contribute to thrombosis through release of poly-phosphates which amplifies FXI activation by thrombin in the contact pathway [33]. Furthermore, platelet cross-talk with neutrophils is associated with NETs formation, a critical player in FXII activation *in vivo* [45]. Neutrophils also release cathepsin-G that promotes PAR activation and thrombosis [38], and elastase that potentiates platelet aggregation by other agonists, and cleaves TFPI [40]. Neutrophil activation also takes place through the complement cascade, which is activatable by plasmin [37,46]. Here we found out that Boophilin inhibits plasmin- and cathepsin G-induced platelet aggregation, and partially attenuates elastase activity, according to degradation of TFPI *in vitro*. Altogether, these results suggest that Boophilin interrupts the platelet-neutrophil axis associated with thrombosis [35,45], shifting the hemostatic system towards an anti-coagulant phenotype. This contention was verified through testing the effects of Boophilin *in vivo*, using the FeCl_3_carotid artery thrombosis model. FeCl_3_ causes endothelial cell injury, with exposure of pro-hemostatic sub-endothelial matrix leading to thrombus formation [47]. Our experiments demonstrated that Boophilin effectively inhibits vessel occlusion under our experimental conditions with prolongation of the bleeding time. Based on our *in vitro* data, the antithrombotic effect of Boophilin is likely the result of inhibiting multiple targets, including thrombin, FXIa, and enzymes associated with neutrophils and platelets. However, the relative contribution of each enzyme inhibition in the antithrombotic properties of Boophilin *in vivo* remains to be determined. Finally, it is not known whether inhibition of FXIa and thrombin are mediated by the same reactive loops.

While most salivary inhibitors are specific for coagulation factors through high-affinity interactions, why midgut Boophilin behave as a multifunctional, relatively weak inhibitor, with low specificity? One explanation might be that in the midgut, the coagulation and inflammatory reactions are no longer under control of endogenous inhibitors involved in the regulation of proteolytic cascades. We speculate that in its absence, pro-hemostatic/inflammatory pathways are severely unbalanced and activated in the midgut. Plausibly, attenuation of only one enzyme in the midgut would not suffice to prevent inflammation and clot formation, which may impair the intracellular digestion through acidic phagolysosomes [10,48]. Salivary thrombin inhibitors found in *R*. *microplus* may also contribute to lower the procoagulant tonus at the feeding attachment site [49–51] in response to mechanical injury promoted by distinct mouthparts [52–54]. In conclusion, much redundancy exist in inflammatory circuits; accordingly, blockade of several arms of inflammation/coagulation activation might confer adaptive or survival advantages when compared to single target inhibition.
